# Colonic Ileus, Distension, and Ischemia Due to COVID-19-Related Colitis: A Case Report and Literature Review

**DOI:** 10.7759/cureus.13236

**Published:** 2021-02-09

**Authors:** Danial H Shaikh, Harish Patel, Jasbir Makker, Kanthi Badipatla, Sridhar Chilimuri

**Affiliations:** 1 Gastroenterology, BronxCare Health System, New York, USA

**Keywords:** sars-cov-2, colostomy, covid 19, colitis, bowel ischemia, gastrointestinal obstruction, tocilizumab, convalescent plasma, gastrointestinal surgery, pseudo-colonic obstruction

## Abstract

Coronavirus disease 2019 (COVID-19) predominantly presents with respiratory symptoms, however, the involvement of the gastrointestinal system has also been reported. Isolated gastrointestinal manifestation due to COVID-19 presenting as colonic distension is uncommon. Colonic ileus from COVID-19 infection presents as dilatation on imaging, with the risk of subsequent ischemia and perforation if not recognized and treated promptly. There is no consensus on the treatment modality for COVID-19-related colitis, however, COVID-19-targeted medications in conjunction with surgical intervention have been performed for management.

We present a case of a 73-year-old man who presented with abdominal pain, distention, and diarrhea. He tested positive for severe acute respiratory syndrome coronavirus 2 (SARS-CoV-2) and was found to have marked dilatation of the colon on imaging. He was initially given convalescent plasma to reduce inflammatory markers, as tocilizumab was contraindicated due to suspected bowel obstruction. Once more stable, he underwent surgical intervention followed by tocilizumab infusion. Pathological specimens of the colon demonstrated hemorrhagic colitis with microthrombi suggestive of COVID-19-related colitis.

Recognizing COVID-19-related colitis allows for timely diagnosis and management with targeted interventions in addition to surgery, which may prevent perforation. We suggest convalescent plasma followed by the formation of colostomy and finally infusion of tocilizumab as a feasible option for the treatment of COVID-19-related colitis. However, further research is needed in order to fully understand this entity and provide guidance for its management.

## Introduction

Severe acute respiratory syndrome coronavirus 2 (SARS-COV-2) is a worldwide pandemic and has infected over 50 million people globally, claiming over one million lives at the time of this writing. The disease caused by this virus is called coronavirus disease 2019 (COVID-19) and predominantly presents as a respiratory tract infection with fever, cough, shortness of breath, with the most severe presentation manifesting as acute respiratory distress syndrome.

Over the course of time, it has become increasingly evident that the gastrointestinal (GI) system can be involved. In fact, the outbreak of severe acute respiratory syndrome (SARS) in 2002-2004 caused by the SARS-COV-1 virus had also depicted gastrointestinal (GI) signs similar to the current pandemic [1].

Studies have suggested that GI symptoms may be present in up to 26% of patients [2]. A meta-analysis involving over 4000 patients with COVID-19 from six countries showed a pooled prevalence of GI symptoms in 17% of patients. The most common GI symptom reported was a loss of appetite followed by diarrhea, nausea or vomiting, and abdominal pain [3]. For many patients with COVID-19, GI complaints may be the only presenting sign of the disease, occurring before the onset of fever or respiratory symptoms. Some reports have described GI symptoms in COVID-19 even in the complete absence of respiratory symptoms. The first known COVID-19 case in the USA was from Washington State and the patient had a two-day history of nausea, vomiting, and loose stool prior to the onset of respiratory symptoms [4].

SARS-CoV-2 primarily gains entry into host cells via the angiotensin-converting enzyme 2 (ACE2) receptor. This receptor is expressed in alveolar cells; however, it is also present in abundance within the epithelial cells of the GI tract. In a study, SARS-COV-2 culture yield from the small intestine was higher than that of specimens obtained from lung tissues, which is generally believed to be the prime target organ of this virus [5]. SARS-CoV-2 may cause digestive symptoms either by direct viral invasion in target cells and/or immune-mediated tissue and end-organ injury. It is known to have a direct cytopathic effect on the colonic mucosa, as evidence by immunofluorescence staining of the GI tract in patients with COVID-19 [6].

It is now common knowledge that COVID-19 is also associated with coagulopathy. The hypercoagulable state is characterized by both microangiopathy and systemic coagulation defects that lead to large vessel thrombosis such as pulmonary embolism. The GI tract is also susceptible to this hypercoagulable state. Bhayana et al. described bowel wall abnormalities in up to 31% of their COVID-19 patients. A few of them required surgical intervention and pathology of surgical specimens revealed ischemic enteritis with patchy necrosis due to fibrin thrombi in arterioles [7]. Reports of acute bowel wall ischemia [8], pneumatosis intestinalis [9], and even colonic perforation [10] due to thromboembolism secondary to COVID‐19 infection exist in the literature.

We present our experience of a patient who presented to our institution with abdominal pain and diarrhea. He tested positive for SARS-CoV-2 but did not have any respiratory symptoms. He was noted to have a colon polyp and colonic distension with no small bowel ileus. He underwent evaluation for possible COVID-19-related colitis leading to large bowel obstruction. We treated him with convalescent plasma in conjunction with surgical intervention for relief of symptoms. Pathological specimens of the colon showed hemorrhage and possible microthrombi. He was later prescribed tocilizumab and eventually made a full recovery.

## Case presentation

A 73-year-old male presented to our emergency department with complaints of severe periumbilical abdominal pain for four to five hours. The patient described the pain as non-radiating, gradual in onset, and progressively worsening. He characterized it as cramping and aching in nature and denied any precipitating or relieving factors. He also complained of associated non-bloody watery diarrhea, five episodes in the same duration. He did not report any nausea or vomiting and denied any hematochezia, hematemesis, or melena.

The patient had the medical comorbidities of hypertension and diabetes for which he was on metformin, carvedilol, and lisinopril. He denied any prior surgeries. He denied smoking, alcohol, or the use of recreational drugs. His family history was non-contributory for colon cancer, and he did not travel anywhere within the past one year. His review of systems was negative for a prior history of abdominal pain or rectal bleeding. He denied any cough, fever, sore throat, chest pain, or shortness of breath.

On presentation, his vital signs demonstrated a heart rate of 81 beats per minute, a blood pressure of 147/69 mmHg, oxygen saturation of 98% on room air, and a temperature of 97.5°F. On physical examination, he was in mild distress from abdominal pain. No skin rashes were appreciated. The cardiopulmonary exam was unremarkable; however, his abdomen was soft with mild distention and tender to deep palpation diffusely. Bowel sounds were normoactive and no guarding, rigidity, or organomegaly was present. The neurological exam was within normal limits.

**Figure 1:**
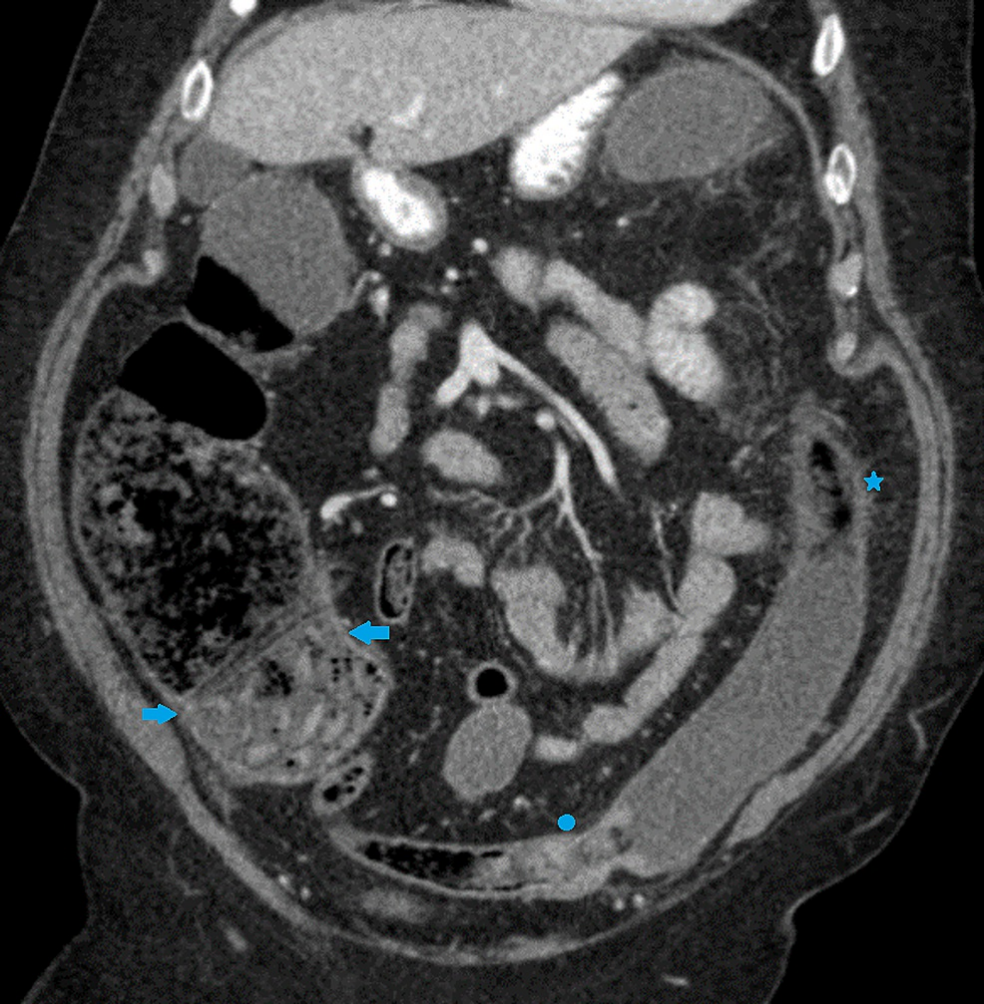
CT scan of the abdomen with contrast on admission demonstrating mild dilatation of the right colon with diffuse wall thickening and surrounding inflammation along the splenic flexure and descending colon CT: computed tomography

Initial laboratory investigations were significant for iron deficiency anemia, an elevated D-dimer, lactate dehydrogenase (LDH), C-reactive protein, fibrinogen, and lactic acid level (Table 1). A computed tomography (CT) scan of the abdomen with contrast (Figure 1) showed mild dilatation of the right colon with diffuse wall thickening and surrounding inflammation along the splenic flexure and descending colon. There was no free fluid. There was no small bowel distension.

**Table 1:** Laboratory values SARS-CoV-2: severe acute respiratory syndrome coronavirus 2; RNA: ribonucleic acid

	On Admission	Day 3	On Discharge
Hemoglobin (g/dL)	9.5	8.8	8.6
White blood cell count (k/uL)	8.7	7.4	3.7
Platelet count (k/uL)	284	331	524
Prothrombin time (s)	10.6	13.4	11.7
INR	0.9	1.1	0.9
Sodium (mEq/L)	137	141	138
Potassium (mEq/L)	4.7	3.7	3.8
Blood urea nitrogen (mg/dL)	29	19	5
Creatinine (mg/dL)	1.1	0.7	0.6
Lactic acid (mmoles/L)	3.4	1.1	-
pH	7.33	7.47	-
Total bilirubin	0.4	-	-
Aspartate transaminase (units/L)	23	-	-
Alanine aminotransferase (units/L)	14	-	-
Alkaline phosphatase (units/L)	90	-	-
Lipase (units/L)	21	-	-
SARS-CoV-2 RNA	Positive	-	Negative
C- reactive protein (mg/L)	286	48	8
Lactate dehydrogenase (units/L)	236	208	217
Ferritin (ng/mL)	60.4	88	39.8
D-dimer (ng/mL)	-	2757	374

**Figure 2:**
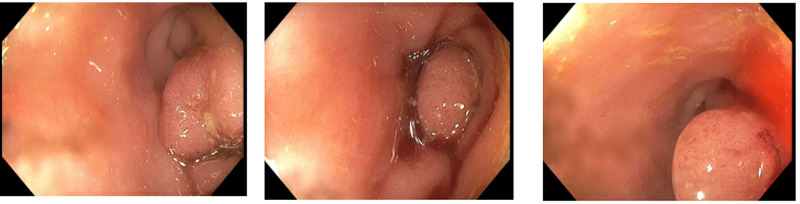
Flexible sigmoidoscopy revealing a polypoid lesion near the proximal sigmoid colon with adjacent ulcerated friable colonic mucosa

He was initially given empiric treatment for Clostridium difficile infection with oral vancomycin and intravenous metronidazole. Sigmoidoscopy was planned for the evaluation of ischemic colitis and to rule out pseudomembranous colitis. The sigmoidoscopy revealed a polypoid lesion near the proximal sigmoid colon with ulcerated friable colonic mucosa (Figure 2) and the scope was not negotiated past the lesion due to the mucosal friability. Pathology of the polypoid lesion revealed fragments of tubular adenoma.

**Figure 3:**
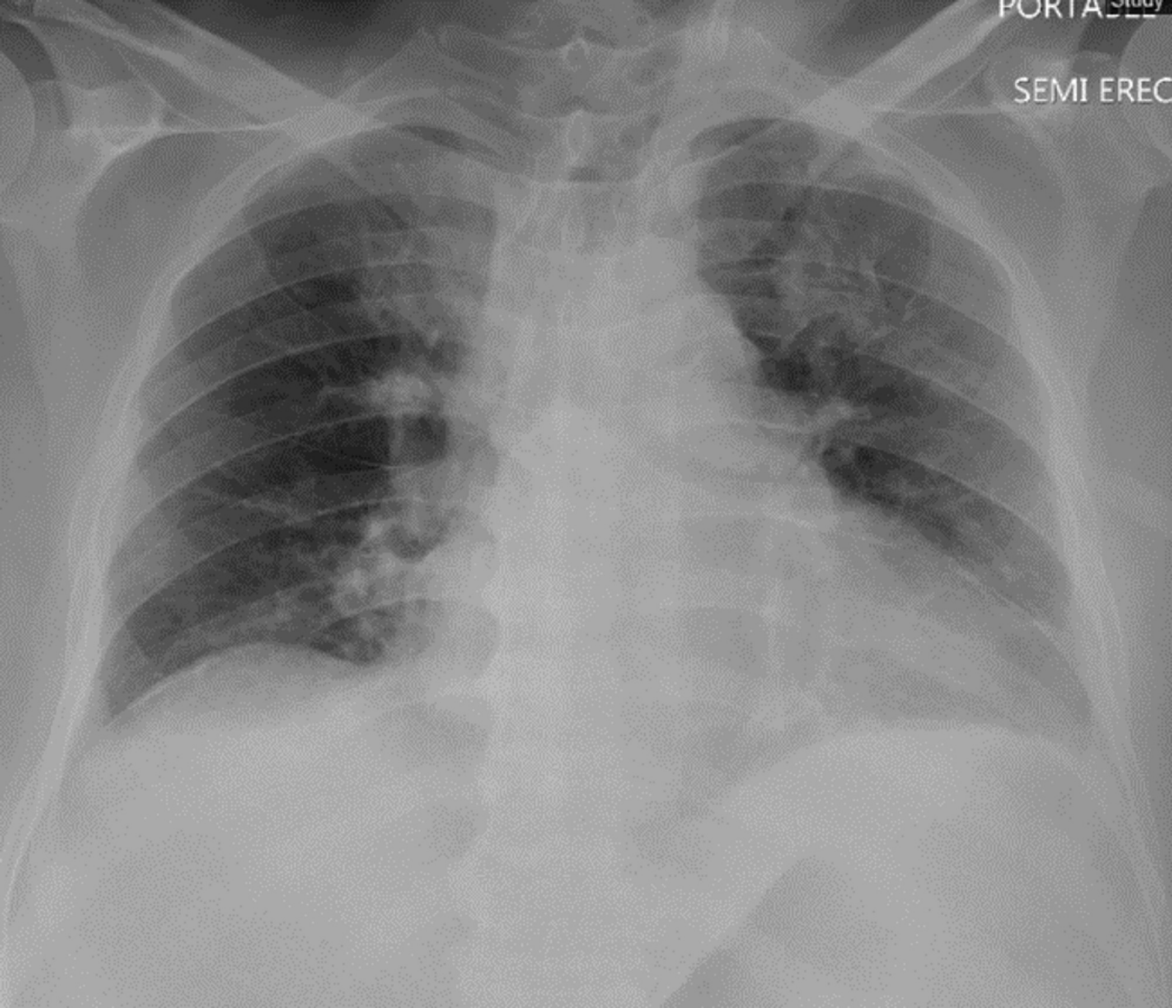
Initial chest X-ray demonstrating a subtle left upper lobe infiltrate

A chest X-ray (Figure 3) revealed a left upper lobe infiltrate, and he tested positive on the nasal swab for SARS-CoV-2. The patient was initiated on COVID-19-targeted therapy. Venous thromboembolism prophylaxis with enoxaparin was provided. Corticosteroids were not given due to suspected Clostridium difficile infection.

**Figure 4:**
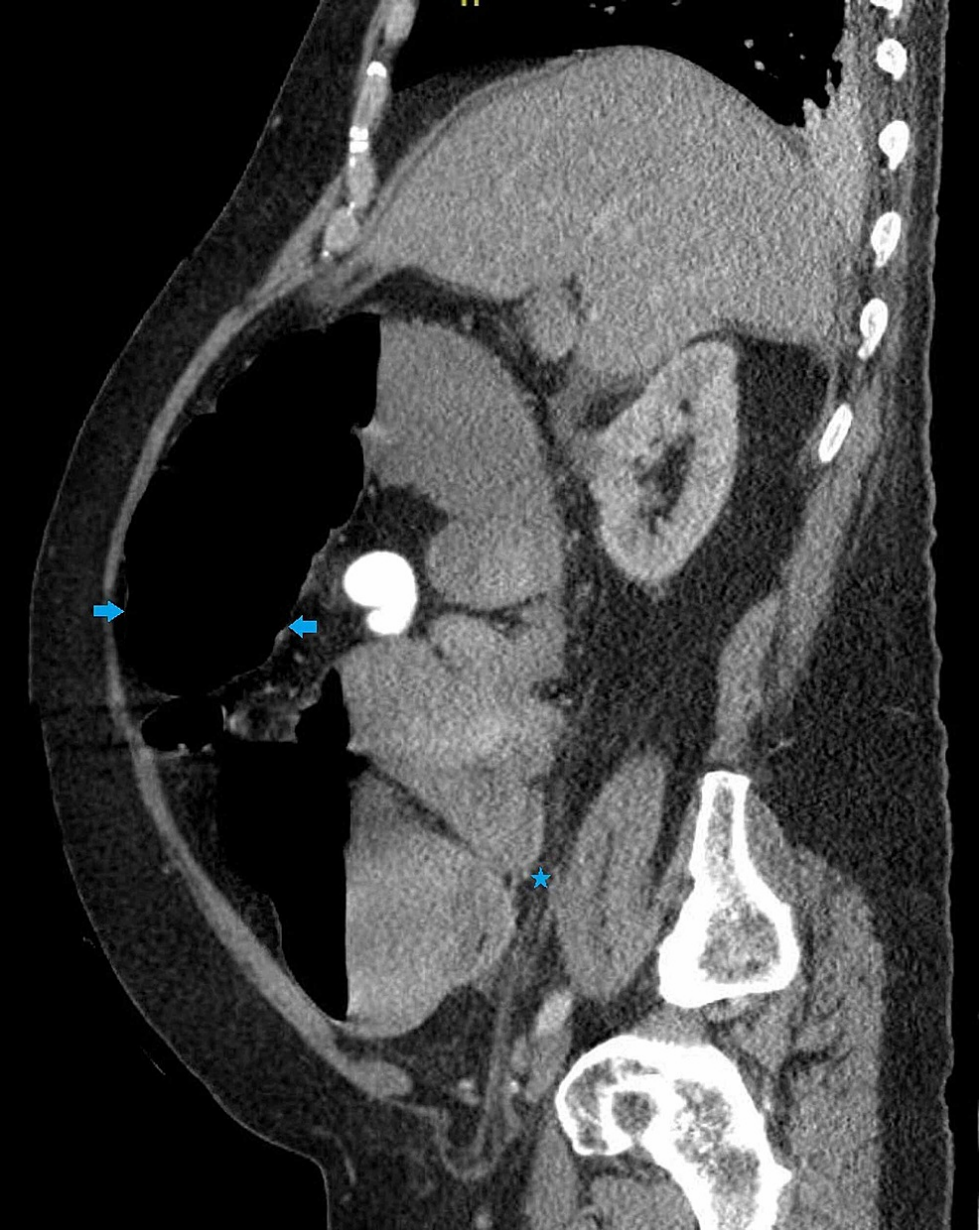
A repeat CT scan of the abdomen demonstrating colonic dilatation and wall thickening involving the distal transverse and descending colon, with pericolonic infiltrative changes representative of inflammatory colitis CT: computed tomography

A nasogastric tube was placed for decompression and a repeat CT abdomen with contrast (Figure 4) was done. The repeat CT showed colonic dilatation of the distal transverse and descending colon, with a maximum diameter of 8 centimeters and wall thickening with pericolonic infiltrative changes representative of inflammatory colitis. The presence of a dilated colon not just proximal but distal to the polyp, as well as a lack of small bowel ileus on imaging, made mechanical colonic obstruction resulting from the polyp less likely. Upon discussion with surgery, COVID-19-related colitis was deemed as the most likely etiology of large bowel obstruction. He was immediately started on convalescent plasma transfusion. Tocilizumab infusion was deferred in view of its potential to cause GI perforation.

Over the course of the day, the patient’s abdominal distention worsened, with an increase in abdominal tenderness. He did not have any bowel movement but was able to pass minimal gas. After discussing with the patient and his family, he underwent transverse loop colostomy, with biopsy of the colonic mucosa from both edges of the colostomy. Pathology of biopsies revealed colonic tissue with marked hemorrhage in the mucosa, and the possibility of vascular thrombi could not be ruled out.

Post-procedure, the patient was transferred to the medical ward where he received 400 milligrams of intravenous tocilizumab in view of the elevated inflammatory markers on presentation. Over the next few days, improvement in his clinical condition and COVID-19-related laboratory indices (Table 1), such as D-dimer, was noted. He was eventually discharged with outpatient gastroenterology and general surgery appointments.

## Discussion

Colonic dilation on imaging studies can be from mechanical large bowel obstruction (LBO) or due to ileus secondary to infectious/inflammatory colitis. LBO is an abdominal emergency with high morbidity and mortality if left untreated. It can lead to mucosal edema, bowel ischemia, and if not treated, bowel infarction, and perforation. Overall, the prevalence of LBO is less common as compared to small bowel obstruction. Earlier in the course of mechanical LBO, colonic dilatation is an isolated finding, however, eventually, the small bowel also becomes involved [11].

Colonic ileus on the other hand is characterized by signs and symptoms of a mechanical LBO in the absence of a mechanical cause. It is characterized by dilatation of the bowel on imaging. There is a growing body of evidence describing large bowel involvement in COVID-19 infection. Colonic ileus secondary to COVID-19 was identified early on in the pandemic. During the peak of the outbreak in Italy, a report identified a COVID-19-positive patient with diarrhea and abdominal pain, who was found to have distension of the large bowel and subsequent perforation of the ascending colon. On laparotomy, neither obstruction of the distal colon nor distension of the small intestine was noted [10].

Kaafarani et al. described GI symptoms in 141 COVID-19 patients in the intensive care unit, of which two underwent exploratory laparotomy for colonic ileus. The pathology of the resected bowel showed focal transmural areas of necrosis with acute fibrinopurulent serositis [12]. Sattar et al. reported three cases of COVID-19 infection with colonic abnormalities on imaging. One of the cases, a 55-year-old man with colonic ileus, had evidence of air in the bowel wall on imaging; however, he did not require surgery and improved with hydroxychloroquine and azithromycin [13]. Ibrahim et al. described two cases of SARS-CoV-2 infection causing paralytic ileus. One of these, a 33-year old man with colonic dilatation underwent emergency laparotomy, which revealed a perforation in the transverse colon. This patient had received treatment with tocilizumab prior to surgery [14]. Other isolated reports have also identified acute ischemic colitis secondary to COVID-19 as a possible etiology of colonic ileus [15].

Ischemic colitis as a consequence of COVID-19 appears to occur in patients presenting with severe respiratory illness. Of the six cases available in the literature, four died as a result of ischemic colitis, and those that did survive either received tocilizumab [16] or underwent the creation of an ostomy in order to prevent perforation [17]. The mechanism for the development of ischemic colitis in COVID-19 seems multifaceted. Shock and hemodynamic instability are common in patients with severe COVID-19, which may result in a decrease in blood supply to the colon. In addition, the use of vasopressors in such patients may lead to significant vasoconstriction of the mesenteric vasculature predisposing to mucosal injury, cellular ischemia, and necrosis. Lastly, the hypercoagulable state of COVID-19 itself may lead to microthrombosis with subsequent ischemia.

The utility of non-surgical SARS-CoV-2 directed interventions like corticosteroids, convalescent plasma, remdesivir, or tocilizumab in patients with colonic ileus is largely unknown. Currently, steroids are recommended only for hospitalized patients with COVID-19, requiring supplemental oxygen. Steroids serve to mitigate the systemic inflammatory response elicited by the SARS-CoV-2. The RECOVERY trial demonstrated mortality benefit in COVID-19 patients who were randomized to receive dexamethasone than those who received the standard of care [18], however, GI symptoms were not evaluated. To date, no studies have looked at the role of steroids on gastrointestinal symptoms in COVID-19 patients. Hence, the safety of steroids in patients with COVID-19 colitis is unknown. If steroids are being considered, all efforts to rule out infectious colitis, such as Clostridium difficile colitis, should be made.

Plasma from donors who have recovered from COVID-19 may contain antibodies to SARS-CoV-2 that may help suppress the virus and modify the inflammatory response. Save for the allergic or anaphylactic transfusion reactions, no adverse GI effects of convalescent plasma have been reported. Hence, it may be an option for the treatment of COVID-19-related colitis. Tocilizumab is a recombinant humanized anti-Interleukin-6 receptor monoclonal antibody. Interleukin-6 (IL-6) is a pro-inflammatory cytokine, and its release is heightened in COVID-19 infection. Thus, modulating the levels of IL-6 with tocilizumab may alter the course of the disease. However, tocilizumab is contraindicated in patients with intestinal obstruction, as there is an increased risk of perforation [19].

Options for surgical intervention for relief of symptoms of colonic ileus include the formation of a loop colostomy or a Hartmann's procedure, with the former having a shorter operative time. Efforts to avoid the laparoscopic approach for procedures should be made, as there is a theoretical risk of occupational exposure from pressurized aerosols during insufflation and longer operative times (and therefore increased risk of exposure) [20].

## Conclusions

It is important to institute SARS-CoV-2 precautions in patients who present solely with GI symptoms. In patients with COVID-19, the management of acute abdomen remains challenging. Recognizing colonic ileus, distention, and ischemia as a possible complication of COVID-19 is paramount. Not only will it allow for a timely diagnosis but can possibly deter consequences such as perforation, which require prolonged surgical intervention (such as total colectomy) and therefore increase the risk of exposure to SARS-CoV-2. Judicious use of plasma and cytokine inhibitors allows us to stabilize patients before more definitive surgical therapies. Sequencing these therapies is important, as some therapies given at the wrong time may lead to worsening of the clinical condition.
